# Do knowledge infrastructure facilities support Evidence-Based Practice in occupational health? An exploratory study across countries among occupational physicians enrolled on Evidence-Based Medicine courses

**DOI:** 10.1186/1472-6963-9-18

**Published:** 2009-01-30

**Authors:** Nathalie IR Hugenholtz, Karen Nieuwenhuijsen, Judith K Sluiter, Frank JH van Dijk

**Affiliations:** 1 Academic Medical Center, University of Amsterdam, Department: Coronel Institute of Occupational Health, PO Box 22700, 1100 DE Amsterdam, the Netherlands

## Abstract

**Background:**

Evidence-Based Medicine (EBM) is an important method used by occupational physicians (OPs) to deliver high quality health care. The presence and quality of a knowledge infrastructure is thought to influence the practice of EBM in occupational health care. This study explores the facilities in the knowledge infrastructure being used by OPs in different countries, and their perceived importance for EBM practice.

**Methods:**

Thirty-six OPs from ten countries, planning to attend an EBM course and to a large extent recruited via the European Association of Schools of Occupational Medicine (EASOM), participated in a cross-sectional study.

**Results:**

Research and development institutes, and knowledge products and tools are used by respectively more than 72% and more than 80% of the OPs and they are rated as being important for EBM practice (more than 65 points (range 0–100)). Conventional knowledge access facilities, like traditional libraries, are used often (69%) but are rated as less important (46.8 points (range 0–100)) compared to the use of more novel facilities, like question-and-answer facilities (25%) that are rated as more important (48.9 points (range 0–100)). To solve cases, OPs mostly use non evidence-based sources. However, they regard the evidence-based sources that are not often used, e.g. the Cochrane library, as important enablers for practising EBM. The main barriers are lack of time, payment for full-text articles, language barrier (most texts are in English), and lack of skills and support.

**Conclusion:**

This first exploratory study shows that OPs use many knowledge infrastructure facilities and rate them as being important for their EBM practice. However, they are not used to use evidence-based sources in their practice and face many barriers that are comparable to the barriers physicians face in primary health care.

## Background

Being a physician involves using up-to-date knowledge to deliver the best possible care to patients [1,2]. An important aim of health care is to avoid a so-called care gap: a discrepancy between the processes of care defined as best practice on the basis of high-quality evidence and the health care provided in usual clinical practice [3]. Evidence-Based Medicine (EBM) has been developed as a strategy to meet this challenge and to apply scientific evidence to the medical practice. EBM is the conscientious, explicit and judicious use of current best evidence in making decisions about the care of individual patients [4]. Unlike hospital clinicians and general practitioners, occupational physicians have the special responsibility to take into account the working conditions of their patients, opportunities and priorities for management, and the impact of legislation in the field of occupational health and safety [5]. As occupational physicians need knowledge from many different sources and disciplines, EBM can be a useful strategy for them [6]. However, persistent barriers in the implementation of EBM for both clinicians and occupational physicians remain [7]. Physicians report a lack of time to practice EBM, they face an ever-growing quantity of scientific evidence that is not easily accessible, and especially older physicians find it hard to acquire EBM skills [7-9].

Knowledge management is currently making its entrance into the medical world and can reinforce the EBM practice of physicians. Knowledge management is a planned approach to collecting, evaluating, integrating, sharing, and improving knowledge, and generating value from it. In the occupational health field, knowledge management can provide an effective and efficient way of organizing what is known and, by using this, improve the quality of occupational health care. Although technology has improved the ability to collect, analyze, and share knowledge rapidly, it has also produced fragmentation of information and systems that are not well integrated. This challenges practitioners' ability to use existing knowledge to advance occupational health practices. Therefore, knowledge management needs to be supported by a knowledge infrastructure so that the right information can be delivered to the right person and place at the right time [10-12]. A knowledge infrastructure includes organizations and institutions in the public – and sometimes also private – sector whose role is the production, maintenance, distribution, and protection of knowledge, e.g. research councils, institutions of higher education, libraries, databases, legal and administrative regulations to support the well-functioning of these entities.

Grimshaw (2004) distinguishes potential push, pull, and linkage and exchange components for an effective knowledge infrastructure. Examples of push components are: help, advice, and information services, practice guidelines, clearing houses for evidence-based tools and knowledge sharing networks. Training in critical appraisal can be seen as a pull component. Local research and development initiatives to identify research priorities and to support local quality improvements can be seen as a linkage and exchange component [13].

As occupational physicians need knowledge from many sources and disciplines to practice EBM, they may benefit from a well-organized knowledge infrastructure to successfully gain access to the required knowledge. Expanding on the findings of Grimshaw (2004), we tried to distinguish elements in the knowledge infrastructure for occupational health. These elements can be regarded as clusters of key facilities that need to be available and that have to be of good quality to support practice. We distinguished four specific elements: (1) education and training, (2) research and development, (3) knowledge products and tools, and (4) knowledge dissemination and access facilities [14].

Elaborating on these four elements, and starting with the first, education and training facilities include basic professional training and continuous professional education, which should be based on the latest body of evidence based on good quality research. One aspect is training in EBM strategies which are necessary to guarantee the use of up-to-date evidence in occupational health care. Research and development include research activities on occupational health and safety by national and regional scientific institutes, universities, professional associations, and private research and development organizations. These activities lead to the production of new knowledge, knowledge products, and tools that can contribute to the evaluation and innovation of health practices. Subsequently, there is a need for custom-made knowledge products conceived as purposefully developed prescriptions or recommendations for practice. Examples of these are: threshold limit values, practice guidelines, protocols for measurement and for evaluation. These products combine scientific evidence with e.g. practical experiences, and often also with legal, economical, ethical, and cultural constraints. They can be interpreted as forms of translation of specific research evidence into practice with the aim of being more directly applicable. Finally, concrete storage, access, and dissemination facilities for knowledge and knowledge products are needed that can be found in (digital) libraries, literature databases, clearing houses, high-quality evidence-based websites, journals, and helpdesks for professionals.

Knowledge infrastructure facilities can be arranged on the local, national and international levels. Locally, a technical infrastructure is needed, such as internet access at the workplace in a company or occupational health service. On a national level, ministries of Labour and of Health Care, national institutes for occupational health and safety, occupational or public health departments at universities, and professional organizations, are key actors. On the international level, key institutions can be identified such as the International Commission on Occupational Health (ICOH), the World Health Organization (WHO), the International Labour Organization (ILO), and the Occupational Health Field in the Cochrane collaboration.

The presence and quality of a knowledge infrastructure is thought to affect the practice of EBM in occupational health care. However, to set priorities and to define concrete objectives for improvement, we need to know more about the impact of the presence and importance of various knowledge infrastructure facilities for EBM practice. Therefore our research question is: "Which contemporaneous evidence-based information do occupational physicians access to guide their evidence-based practice, and what are the enablers and barriers to them practising EBM?" Subsequently, in this study we explore the knowledge infrastructure in an international approach as we perceive many advantages in the development of an international perspective and in new initiatives fostering international collaboration. The study aims to explore what facilities in the knowledge infrastructure are used and are perceived as important by occupational physicians who are enrolled on EBM training courses in different countries in their EBM practice. Secondly, it aims to explore which (evidence-based) sources OPs use to solve their cases. Finally, the study aims to inventorise the enablers and barriers that OPs experience when practising EBM.

## Methods

### Study population

We invited OPs who planned to attend a course in EBM to participate in our study. An advantage of this approach would be that these OPs, potentially innovators in practising EBM in their country, might already have practical experiences in the use of various facilities and have a good overview of the available knowledge infrastructure. We recruited schools and training centres in occupational medicine via our own contacts, contacts of our colleagues at the Coronel Institute of Occupational Health, and via the European Association of Schools of Occupational Medicine (EASOM). As a result, we approached national postgraduate education and training centres in 14 countries and asked them if they were preparing a course in EBM in the period from August through November 2007 and were willing to collaborate in our study. If so, they were asked if they were willing to recruit potential participants for our study. If a potential participant was interested, he or she could (voluntarily) give his or her email address to the teacher. The teachers provided us with those email addresses and thereafter we contacted these potential participants by email with detailed information on our study, including an informed consent form. Those participants who sent back their signed informed consent form were sent a questionnaire on the knowledge infrastructure.

### Study design and outcome measurements

In a cross-sectional study design, an electronic questionnaire on participants' characteristics, the use and importance of knowledge infrastructure facilities, the use of sources to solve cases in daily practice, and perceived enablers and barriers in practising EBM was used. Participants' characteristics included questions on country, gender, age, education, and previous training in any kind of epidemiology or EBM.

#### Use and importance of knowledge infrastructure facilities

We assessed the use of infrastructure facilities and the participants' perceived importance of these facilities for EBM practice. We developed a questionnaire containing questions on the four specific elements that we regarded as key facilities needed to support EBM practice: (1) education and training, (2) research and development, (3) knowledge products and tools, and (4) knowledge access facilities.

OPs were asked to what extent training in EBM was a sufficient part of their basic medical curriculum, vocational training, respectively postgraduate training. Furthermore, they were asked to judge the importance of being trained in EBM during the three subsequent educational periods. These items were scored on a ten-point scale ('0': "no part at all/not important at all" till "10": "very big part/very important).

In the case of the research and development element, the participants were asked if they were able to use facilities from national or local knowledge centres, research and development institutes, or universities on a dichotomous scale (yes, no). The importance of these facilities for EBM practice had to be rated on a ten-point scale ('0': "not important at all" to "10": "very important) and were sequentially transformed into a score between zero and one hundred. Furthermore, the OPs were asked if the institutes were involved in developing knowledge products and tools, and in education and training. These scores were scored on a dichotomous scale (yes, no).

For the knowledge products and tools, the participants were asked about the use and importance of threshold limit values, practice guidelines, measurement or health care protocols, and criteria documents. The use was scored on a dichotomous scale (yes, no), the importance on a ten-point scale ('0': "not important at all" to "10": "very important). These scores were transformed into a score between zero and one hundred.

In the case of the knowledge access facilities, participants were asked about the use and importance of: information centres or helpdesks; traditional libraries; online or web-based libraries; medical literature databases; occupational health literature databases; full-text articles; google/yahoo/etc; professional web-based forums or communities; and 'question-and-answer' facilities. The use of knowledge access facilities was scored on a dichotomous scale (yes, no), the importance on a ten-point scale ('0': "not important at all" till "10": "very important) and the last score were transformed into a score between zero and one hundred.

#### Use of sources to solve a case

The use of sources to solve cases in daily practice was assessed on three items, by using the 'Reading and evidence-seeking behaviour' part of the questionnaire of Taylor et al. (2004) [15].

The OPs reported the number of hours spent on solving a specific case, and the number of articles read to solve a specific case over the previous month. In addition, they were asked to report on the proportion (%) of these articles that they read thoroughly, skimmed, or read only the abstract of. Finally, the OPs reported how often they used specific sources to solve their cases: review articles in international journals, original research reports in international journals, national journals, textbooks, internet resources/computer databases or similar, guidelines, the Cochrane Library, and colleagues. The frequency of the use of these sources to solve a case was reported on a five-point Likert scale (0: never, 1: rarely, 2: occasionally, 3: often, 4: very often). We reported the percentage of OPs that reported using a source 'often' or 'very often'.

#### Enablers and barriers in practising EBM

OPs were asked to rank their self-formulated top three of enablers, respectively barriers, in practising EBM. We reported the six most frequently mentioned enablers and barriers by the OPs. Furthermore, we asked the OPs to rate the support they receive for practising EBM on a ten-point scale.

### Analysis

We described all outcome measures by means of descriptive analyses. All analyses were carried out using SPSS 13.0.

## Results

### Baseline characteristics

In total, 89 OPs returned an informed consent form and 36 OPs returned a completed questionnaire on the knowledge infrastructure (response rate: 40%).

OPs came from Italy (8), the Czech Republic (6), South Africa (6), Austria (4), Japan (4), Far East (4), Croatia (2), Colombia (1), and Greece (1). About 60% of them are younger than 40 years of age and 17 OPs are male compared to 19 female OPs. Half of the OPs were still in training and two-thirds of the participants had completed an epidemiology training. Only one-quarter had completed a training in EBM.

### Knowledge infrastructure

For an overview of the knowledge infrastructure and the perceived importance of EBM practice, four figures are presented on the conceived elements of the knowledge infrastructure that we believe are required to support Evidence-Based Practice (EBP).

**Figure 1:**
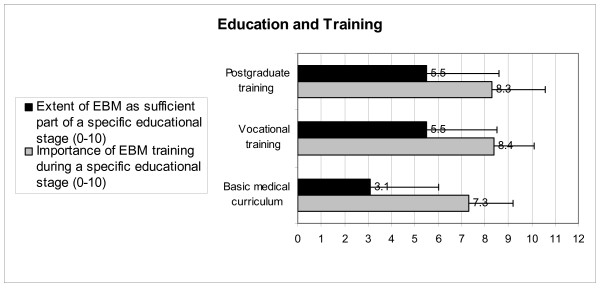
**Extent of sufficient EBM training and the perceived importance of EBM training during OPs' basic medical curriculum, vocational training, and postgraduate training**.

Figure 1 illustrates that, on average, EBM is not thought to be a sufficient part of the basic medical curriculum, but about half of the OPs mention that there is sufficient EBM training in the vocational or postgraduate stage. In general, OPs consider EBM training during the basic medical curriculum as being important and even more important during their vocational and postgraduate training.

**Figure 2:**
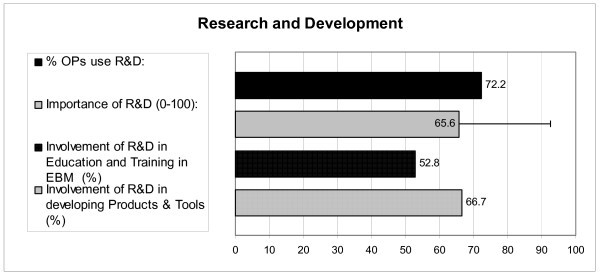
**The use, importance, and involvement of national or local knowledge centres, research and development institutes and universities**.

Figure 2 shows the role the national or local knowledge centres, research and development institutes and universities play in the infrastructure. These Research and Development centres, institutes and universities are quite important for the EBP of the OPs. Almost three-quarters of the OPs are able to use facilities provided by these R&D institutes. OPs state that the institutes are to a large extent involved in developing knowledge products like threshold limit values or practice guidelines (66.7%) and to a slighter less extent involved in the education or training in EBM (52.8%).

**Figure 3:**
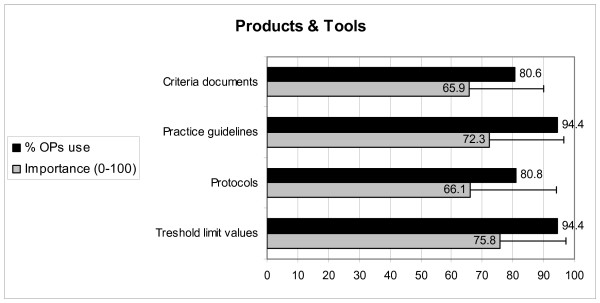
**The percentage of OPs that use knowledge products and tools, and their average importance, according to OPs**.

Figure 3 presents the use of knowledge products and tools by OPs, as well as the perceived importance of the products and tools for evidence-based practice. The figure shows that the large majority of OPs use knowledge products or tools in their practice, especially threshold limit values and practice guidelines. The products or tools are considered equally important. Additionally, OPs report that most of these products or tools are available at the OPs' own workplace or at their company.

**Figure 4:**
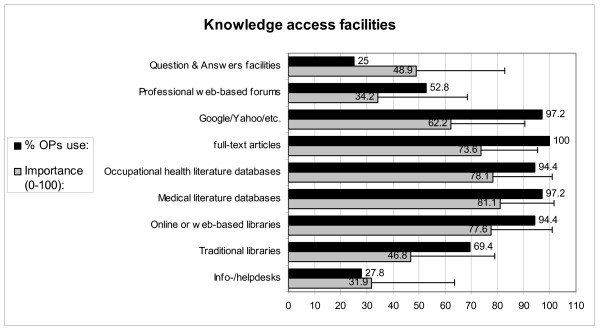
**The use and importance of knowledge access facilities according to OPs**.

Figure 4 shows that search engines like Google or Yahoo, full-text articles, occupational health literature databases, medical literature databases, and online or web-based libraries are used by almost all OPs. These knowledge access facilities are also rated as being most important for evidence-based practice, with the exception of Google/Yahoo, which had a lower rating, while the importance of traditional libraries is also rated relatively low. Only a quarter of the OPs use Question-and-Answer facilities and information- or helpdesks. However, the OPs' mean rate of the importance of the question-and-answer facilities is rather high: 49 points.

### Use of sources

To solve a case during the previous month, OPs use on average four articles and spent more than five hours reading professional literature (Table 1). In half of the cases they read only the abstract. Internet, colleagues, and guidelines are the sources most frequently used, while the Cochrane library, original research in international journals and national journals are the sources least used.

**Table 1:** Source use during EBM practice by occupational physicians (N = 36).

	**Mean score (SD)**
**Number of journal articles looked at or read thoroughly last monthto solve a case**	3.9 (6.4)

**Number of hours spent reading professional literature last month to solve a case**	5.6 (7.8)

**Proportion (%) of the articles read**	

Read thoroughly	20%
Skimmed	25%
Read abstract only	45%

**Types of source used often or very often by OPs (%) to solve a case**	

International journals: review articles	41.6%
International journals: original research reports	22.3%
National journals	25.0%
Textbooks	50.0%
Internet resources/computer databases	69.4%
Guidelines	58.3%
The Cochrane Library	16.7%
Colleagues	63.9%

### Enablers and barriers in EBM practice

The OPs rate the support they receive to practise EBM with 6 points, on average (ten-point scale, SD: 3.16). The most important enablers for practising EBM are medical literature databases, (online) occupational health literature database, online libraries, and full-text articles (table 2). The main barriers are lack of time, payment for access to full-text articles, language barrier (most texts are in English), lack of support and limited EBM skills, and unreliable internet connectivity.

**Table 2:** Occupational physicians' six most important enablers and barriers for practising EBM.

**Enablers**	**Barriers**
1. Medical literature databases	1. Lack of time
2. Occupational health literature database	2. Payment for full-text articles
3. Online libraries	3. Language difficulties
4. Full-text articles	4. Lack of support
5. Traditional libraries	5. Lack of skills
6. Continuing education	6. Unreliable internet connectivity

## Discussion

This study aimed to explore what facilities in the knowledge infrastructure are used and are perceived as being important by occupational physicians (OPs) across different countries, which sources are being used to solve a specific case, and which enablers and barriers OPs experience when practising EBM. The results showed that education and training, and research and development institutes are used by most OPs and are rated as important, but education and training in EBM during the basic medical curriculum can be improved. A variety of products and tools are used often and rated as being important for EBM practice. In knowledge access facilities, more differences can be distinguished. It seems that traditional knowledge access facilities like traditional libraries are still being used often, but are becoming less important. Conversely, novel knowledge access facilities, like question-and-answer facilities, are not (yet) being used very often, but are rated as being quite important facilities for EBM practice by the OPs. To solve their cases, OPs mostly use less evidence-based sources. They prefer the internet, colleagues, and textbooks to solve cases. However, the kind of (evidence-based) sources that they reported not using very often, e.g. the Cochrane library, original research in international journals, and information in national journals, are regarded as important enablers for practising EBM. The main barriers to practising EBM are lack of time, payment for full-text articles, the language barrier, and lack of skills and support.

### Strengths and weaknesses of the study

This study is a first attempt to describe the knowledge infrastructure for OPs across different countries. Unfortunately, the response rate was low (40%), including 36 out of 89 OPs in the study. The language of the questionnaire (English) might have been a difficulty, since nearly all participants were non-native English speakers. In addition, since the OPs were probably occupied by their EBM course, filling out the questionnaire was too time-consuming.

This study informs us about the knowledge infrastructure available for, and valued by, a select sample of OPs already planning an EBM course and who presumably can be regarded as innovators or pioneers in practising EBM. The advantage of this is that they can be considered to be local opinion leaders who can successfully promote EBM. Knowledge about their perceptions might be crucial for progress in EBM practice within occupational health care [16]. However, by selecting participants through educational institutes, some knowledge infrastructure facilities were perhaps provided through that institute. This could differ from the use and perceived significance of OPs not connected with an educational institute. Furthermore, by selecting participants through educational institutes another source of selection bias might be the possibility that our sample of OPs is younger and/or less experienced, or that training in EBM has been identified as part of personal learning needs. In addition, our demographic data showed that 50% were still in training, so inexperience may be a critical factor. Considering these limitations, it should be taken into account that the generalisability of our study results is low.

### Relation with other studies

To solve a case, OPs most often used sources such as the internet, colleagues, and textbooks. These sources are similar to the ones Schaafsma et al. (2004) found [17]. However, these sources are not considered to be the most evidence-based sources. There is a chance that the information is of lower quality as it has been proven – in both clinical and occupational health settings – that advice physicians receive in their daily practice, e.g. from colleagues, differs substantially from the best available evidence from literature [18]. The frequency of use of knowledge sources in our study is comparable with the findings of Taylor et al. (2004) whose 'evidence-seeking behaviour' scale we used. In this randomized controlled trial among 145 general practitioners, hospital physicians and other health professionals, the Cochrane Library was used least frequently. The use of all other sources of their respondents was similar to the finding in our study, except for the use of internet resources. OPs in our study use the internet substantially more often compared to the respondents in the study of Taylor et al. [15]. In this study, lack of time, lack of EBM skills, and payment for full-text articles were considered to be the most important barriers. The first two barriers correspond with findings of various recent studies in both primary health care and occupational health care [7,9,17,19].

### Possible mechanisms and implications

According to the OPs, training in EBM was not a sufficient part of the basic medical curriculum of the OPs. As three-quarters of the OPs were 30 years or older, it is likely that they completed their basic medical curriculum at least five years ago. We have to take into account that EBM was not yet a well integrated part of the basic medical curriculum in many universities at that time. EBM has only existed for about the last 20 years, and was introduced into the medical curriculum in the last decade in most countries [20-22]. For many health professionals, EBM is therefore being taught in vocational and postgraduate courses. Most likely, currently EBM is part of the basic medical curriculum to a larger extent, and will perhaps expand in the next years.

Knowledge products and tools, like threshold limit values, protocols, guidelines, and criteria documents, are being used by most of the OPs. This implies that OPs are familiar with using forms of consolidated knowledge in practical instruments which are easy to apply in daily practice [23].

To access knowledge, OPs mostly use web-based libraries, medical or occupational health literature databases, and search engines like Google or Yahoo. Professional web-based forums or communities and Question-and-Answer facilities are not frequently used by OPs. However, these facilities are relatively new. Their use may be expended in the near future since more of these kinds of facilities are being developed and offered. Lack of EBM skills and payment for full-text articles were considered to be important barriers.

Hopefully, EBM practice will improve over the years as the new generation of OPs will probably receive EBM education during their basic medical education. Furthermore, evidence on effective methods for EBM teaching is growing, and new and better approaches for EBM education are being developed [24-26]. Since OPs consider medical and occupational health literature databases and full-text articles as the most important enablers for practising EBM, it is regrettable to conclude that most OPs do not have cost-free access to these articles. By demanding high fees for full-text access, publishers maintain their exclusive position in disseminating scientific evidence. In addition, a few databases that are essential for occupational health professionals charge for the use of their databases. Fortunately, there is a special arrangement for low- and middle-income countries. The Health InterNetwork Access to Research Initiative (HINARI) programme, set up by the WHO in collaboration with major publishers, provides free or extremely low-cost online access to the major journals in biomedical and related social sciences to local, not-for-profit institutions in developing countries [27]. Access to the Cochrane Library, which was the source used least often by our participants, is also steadily improving for low-income and middle-income countries, as institutional and national subscriptions become more common. In several industrialized countries, government grants enable citizens to use the library at no cost [28]. Especially since the recent introduction of the occupational health field in the Cochrane Collaboration, this is encouraging for the enhancement of EBM practice by OPs [29].

## Conclusion

To ensure high professional quality, EBM practice by OPs is essential and a high-quality level of the knowledge infrastructure can support this. However, simply enabling (local) access to knowledge may not be sufficient to effectively change EBM practice [30]. Other aspects which are important for the support of EBM practice have to be taken into account as well. Support from colleagues and management to practise EBM is important, as well as the motivation of OPs to take responsibility for delivering the best possible occupational health care. New initiatives in providing cost-free access to medical and occupational literature databases and full-text articles may contribute to a better knowledge dissemination. Further research to explore the impact of these aspects on EBM practice among OPs and other occupational health professionals can support innovations in practice. Further research on knowledge infrastructure facilities related to OPs practicing EBM should include a wider sample than the population in this study. More OPs per country and per work setting could be included to make comparison of differences in knowledge infrastructure facilities on national (countries) on local (work settings) level possible. Moreover, not only the OPs who can be regarded as innovators should be included. It would be interesting to know which knowledge infrastructure facilities are available for the more average OPs and what kind of sources they use to solve their cases. Obtaining a sound grasp of the knowledge facilities (not) available for these OPs in diverse countries and work settings can demonstrate the initiatives and improvements needed to support the EBM practice in occupational health care.

## Competing interests

The authors declare that they have no competing interests.

## Authors' contributions

FvD and NH conceived of the study. All authors participated in the design of the study. NH and FvD coordinated the study. NH carried out the study and collected the data. NH and KN performed the analysis and NH drafted the manuscript. All authors read and approved the final manuscript

## Pre-publication history

The pre-publication history for this paper can be accessed here:


